# A FBWM-PROMETHEE approach for industrial robot selection

**DOI:** 10.1016/j.heliyon.2020.e03859

**Published:** 2020-05-08

**Authors:** Mahdi Nasrollahi, Javaneh Ramezani, Mahmoud Sadraei

**Affiliations:** a Imam Khomeini International University (IKIU), Faculty of Social Sciences, Qazvin, Iran; b NOVA University of Lisbon, Faculty of Sciences and Technology and UNINOVA-CTS, Campus da Caparica, 2829-516, Monte Caparica, Portugal; c Business Consultant, Sydney, Australia

**Keywords:** Industrial engineering, Multidisciplinary design optimization, Manufacturing engineering, Technology management, Operations management, Industry management, Business management, Industrialization, Industrial robots, Fuzzy best-worst method, PROMETHEE, MCDM, Robot selection, Criteria

## Abstract

In recent years, the selection of a robot for particular industrial purposes is one of the most challenging problems in the manufacturing environment based on automation and smartness for real-time decision-making. At present, several types of industrial robots with various capabilities, features, facilities, and specifications are available in the market. This makes the decision-making process more and more complicated due to the increase in complexity, advanced technologies, and features that are continually being incorporated into the robots by several manufacturers. The decision-maker needs to identify and select the best-suited robot to attain the desired output with precise application ability, and minimum cost. This paper tries to solve the robot selection problem using Fuzzy Best-Worst Method and PROMETHEE as the two most appropriate multi-criteria decision-making (MCDM) methods for weighting criteria and ranking of decision alternatives, respectively.

## 1 Introduction

Nowadays, the utilization of robots with distinct capabilities, features, and specifications has increased massively because of the developments in information technology and engineering. Robots' features are playing critical roles in today's industries. A robot is usually self-control, multipurpose, reprogrammable machine (Athawale and Chakraborty, 2011; Chatterjee et al., 2010; Rao, 2007). These features make the robot an essential tool to perform a variety of tasks in diverse industrial applications, including material handling, assembly, finishing, machine loading, spray painting, and welding. Moreover, organizations have increased their productivity by using robots. The implementation of IT by organizations is associated with such advantages as improved operation speed, increased reliability in the production process, improved quality, etc. Additionally, in today's competitive market, companies have realized the importance of selecting proper machines that can perform their requirements with the desired quality and within a scheduled timeframe. One of the critical challenges faced by the managers for maintaining the competitive advantage is the selection of strategic machines and robots effectively.

According to Kumar, E. S. R. R., & Prasad, J. S. R. (2018), “the objective of a robot selection procedure is to identify the robot selection attributes and obtain the most appropriate combination of the characteristics in conjunction with the real requirements of the industrial application. A robot selection attribute is defined as a factor that influences the selection of a robot for a given industrial application”. These attributes affecting the robot selection decision can be classified as beneficial and non-beneficial attributes. The beneficial attributes are desired in higher values, e.g., load-carrying capacity, programming flexibility, by contrast, non-beneficial characteristics are those that preferably expected to be in lower values, e.g., cost and repeatability (Kumar, E. S. R. R., & Prasad, J. S. R., 2018). Recent emphasis has been placed on many important strategic attributes such as maximum tip speed, memory capacity, and supplier's service quality, purchasing cost, repeatability, and flexibility, etc. (Rao, 2007; Rao et al., 2011; İç et al., 2013; Kumar and Garg, 2010; Parameshwaran et al., 2015) consideration while selecting an industrial robot for a particular application. Robot selection for a specific application and production environment from among a large number of available options in the market has become a difficult task. Besides, robots are still a somewhat new concept in the industry, and then it is not unusual for an enterprise to be a first-time robot purchaser. With this trend in mind, developing a process for evaluating and ranking of robots to select the best robot seems a necessity.

In such a case, many precision-based methods for robot selection have been developed. For instance, Multi-criteria decision-making (MCDM) models, including ELECTRE and VIKOR methods for the selection of suitable robots, are available in Chatterjee et al., 2010. Kumar and Garg (2010) proposed a Distant Based Approach (DBA) for evaluation, selection, and ranking of robots. Rao et al., (2011) reported a novel decision-making method with objective and subjective preferences to assess and rank robots under the Fuzzy environment. İç et al. (2013), proposed a robot selection decision support system (ROBOSEL) to help decision-makers in their robot selection using FAHP for obtaining and arranging an independent set of criteria and ranking the feasible robots. Rashid et al. (2014), designed an applicable method using generalized interval-valued fuzzy numbers with TOPSIS for the selection of robots. Liu et al. (2014), suggested an interval 2-tuple linguistic MCDM method for robot evaluation and selection. In addition, Parameshwaran et al. (2015), used an integrated fuzzy MCDM based approach for robot selection considering objective and subjective criteria. Their approach utilizes Fuzzy Del-phi Method (FDM), Fuzzy Analytical Hierarchical Process (FAHP), Fuzzy modified TOPSIS or Fuzzy VIKOR and Brown–Gibson model for robot selection. Ghorabaee (2016), employed the VIKOR method and Interval Type-2 Fuzzy sets to assess and select robots. Zhou et al., (2018), developed a Fuzzy extended VIKOR-based model for mobile robot selection for hospital pharmacy. Wang, Miao, Cui and Liu (2018), proposed a Robot Evaluation and Selection model with Entropy-Based Combination Weighting and Cloud TODIM Approach. Narayanamoorthy et al., (2019), proposed interval-valued intuitionistic hesitant fuzzy entropy for determining the importance of the criteria and interval-valued intuitionistic hesitant fuzzy VIKOR method for ranking the robots. Sharaf (2018), proposed a novel approach to choose among alternatives, differently assessed by decision-makers on different criteria, to make the final evaluation for decision-making.

As mentioned above, various MCDM methods incorporated into fuzzy theory has been reported in the literature for the robot selection process. However, still, efforts need to be extended to determine influential attributes for a given industrial application and strengthen the existing robot selection procedure using logical approaches. The literature review demonstrates the power of Best-Worst Method (BWM) and Preference Ranking Organization Method for Enrichment Evaluations (PROMETHEE) in addressing MCDM problems (Ramezani et al., 2019). Integrating Fuzzy Best-Worst Method (FBWM), and PROMETHEE approaches, which never has used before in the robot selection problem, seems to be a powerful combination to overcome uncertainties in the decision-making process. Therefore, this paper intends to develop a new decision-making method that takes care of suitable criteria selection and proper evaluation of the alternatives treating it as an MCDM problem. The proposed approach integrates a one-sample t-test, FBWM, and PROMETHEE. To do so, the remainder of this paper is organized as follows. The proposed methodology is explained in Section 2, followed by Section 3, which demonstrates the proposed method in the selection of robots for industrial purposes. Conclusions are presented in Section 4.

## 2 Proposed approach

**Figure 1:**
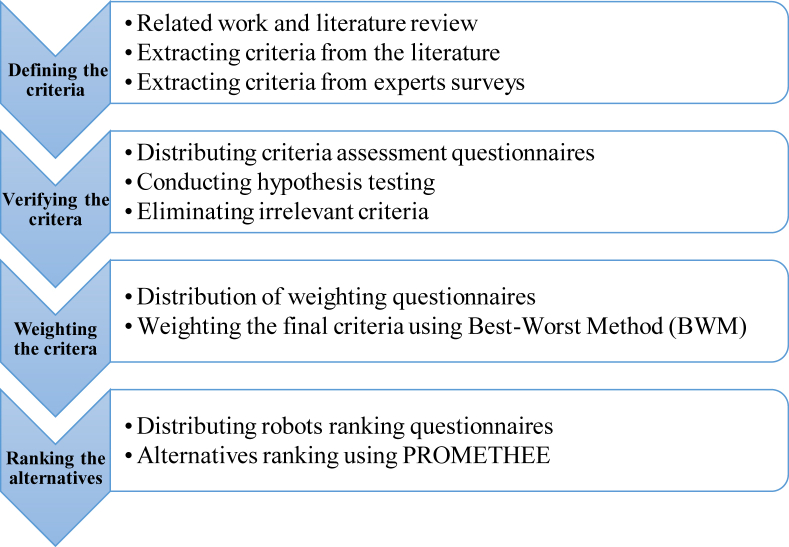
An integrated approach for robot selection.

The general procedure of robot ranking and selection problem has been summarily indicated in Figure 1, which incorporates four main stages. First, the assessment criteria of industrial robots were extracted from the literature review and experts’ survey. In the second stage, to verify the effectiveness of the identified criteria, a questionnaire consisting of 5-point Likert scale questions were distributed among field experts and the final criteria were determined using the one-sample t-test in SPSS software. In the third stage, based on the questionnaires answered by the experts, the final criteria were weighted using the FBWM. In the final stage, the evaluation of alternatives is conducted using the PROMETHEE method. The steps of this research are summarized in Figure 1.

The statistical population of this study consisted of 52 experts who have much experience in the field of automotive, industrial robots.

## 3 Results and discussion

The objective of this study is to develop a procedure combining various robot attributes to enable a comprehensive ranking of alternative robots. Hence, in this section, we present the main findings of the proposed approach regarding critical criteria identification and performing methods to define and evaluate suitable criteria and proper decision alternatives. The proposed approach integrates FBWM and PROMETHEE methods as the two most appropriate MCDM methods for weighting criteria and ranking of decision alternatives.

### 3.1 Critical criteria identification

The process of determining the criteria for the selection of industrial robots was revealed by the literature review. To complete the evaluation criteria for this study, and considering the structural, contextual and environmental differences between Iranian industries in comparison with the other parts of the world, 10 experts were interviewed by telephone and other telecommunication platforms. This was done to ensure that any potentially overlooked criteria would ultimately be identified and added to the list. Finally, given the results obtained by examining the literature and expert surveys, 12 criteria were listed (Table 1) as the initial criteria for the selection of robots. As seen in Table 1, Load capacity, Repeatability, Cost, Velocity ratio, Man-Machine Interface, and Programming flexibility are the most common criteria applied for ranking robots.

**Table 1:** Robot selection criteria.

Criterion	Reference
Cost (CO)	(Karsak et al., 2012; Koulouriotis and Ketipi, 2011; Kumar and Prasad, 2018; Liu et al., 2014; Narayanamoorthy et al., 2019; Rao, 2007; Sharaf, 2018; Talluri and Yoon, 2000; Tao et al., 2012; Vahdani et al., 2014; Wang and Chin, 2009; Zhou et al., 2018)
Load (carrying) capacity (LC)	(Athawale and Chakraborty, 2011; Chakraborty, 2011; Karande et al., 2016; Karsak et al., 2012; Koulouriotis and Ketipi, 2011; Kumar and Prasad, 2018; Kumar and Garg, 2010; Liu et al., 2014; Narayanamoorthy et al., 2019; Rao, 2007; Talluri and Yoon, 2000; Tao et al., 2012; Wang and Chin, 2009; Zhou et al., 2018)
Repeatability (RE)	(Athawale and Chakraborty, 2011; Chakraborty, 2011; Karande et al., 2016; Karsak et al., 2012; Kumar and Prasad, 2018; Kumar and Garg, 2010; Narayanamoorthy et al., 2019; Rao, 2007; Sharaf, 2018; Talluri and Yoon, 2000; Tao et al., 2012; Vahdani et al., 2014; Wang and Chin, 2009; Zhou et al., 2018)
Man-Machine Interface (MMI)	(Koulouriotis and Ketipi, 2011; Kumar and Prasad, 2018; Liu et al., 2014; Narayanamoorthy et al., 2019; Rao, 2007; Sharaf, 2018; Vahdani et al., 2014; Wang et al., 2018; Zhou et al., 2018)
Programming flexibility (PF)	(Koulouriotis and Ketipi, 2011; Kumar and Prasad, 2018; Liu et al., 2014; Narayanamoorthy et al., 2019; Rao, 2007; Sharaf, 2018; Vahdani et al., 2014; Wang et al., 2018; Zhou et al., 2018)
Maximum tip speed (MTS)	(Athawale and Chakraborty, 2011; Chakraborty, 2011; Karande et al., 2016)
Memory capacity (MC)	(Athawale and Chakraborty, 2011; Chakraborty, 2011; Karande et al., 2016) (Narayanamoorthy et al., 2019)
Manipulator reach (MR)	(Athawale and Chakraborty, 2011; Chakraborty, 2011; Karande et al., 2016)
Velocity ratio (VR)	(Karsak et al., 2012; Koulouriotis and Ketipi, 2011; Kumar and Garg, 2010; Narayanamoorthy et al., 2019; Talluri and Yoon, 2000; Tao et al., 2012; Vahdani et al., 2014; Y. M. Wang and Chin, 2009; Zhou et al., 2018)
Degree of freedom (DF)	(Chakraborty, 2011; Kumar and Garg, 2010; Narayanamoorthy et al., 2019)
Vendor's service contract (VSC)	(Liu et al., 2014; Sharaf, 2018; Wang et al., 2018)
Positioning accuracy (PA)	(Liu et al., 2014; Narayanamoorthy et al., 2019; Sharaf, 2018; Zhou et al., 2018)

Given the proposed methodology, although the collected criteria are the results of a literature review and open interviews, at the same time, no particular emphasis was seen by previous studies on the difference between criteria in various industries. The initial criteria were tested using a questionnaire put forward to the field experts. Cronbach's alpha was used to assess the reliability of the questionnaire. Table 2 displays the result of the reliability test. As is shown, the reliability of the questionnaire was higher than 0.7 and, thus, deemed optimal.

**Table 2:** Reliability statistics.

No. of items	Cronbach's alpha based on standardized items	Cronbach's alpha
48	.917	.889

For the next step, the Kolmogorov-Smirnov test was used to determine the distribution of research variables. The results of the K–S test for each primary criterion are displayed in Table 3. Since the significance level of the assessed criteria is higher than 0.05, we can conclude that all criteria are distributed normally and, thus, it is possible to use the parametric test.

**Table 3:** Result of the Kolmogorov-Smirnov test.

Criterion	CO	LC	RE	MMI	PF	MTS	MC	MR	VR	DF	VSC	PA
N	48	48	48	48	48	48	48	48	48	48	48	48
Mean	3.9012	3.8380	3.9710	4.0010	3.8730	3.8920	3.9260	3.9018	3.8451	3.8520	3.8036	3.9236
SD	0.6736	0.6593	0.6549	0.6634	0.6498	0.6602	0.6367	0.6399	0.6796	0.63617	0.6796	0.6538
Z	1.270	1.270	1.504	1.447	1.431	1.526	1.260	1.231	1.489	1.270	1.489	1.269
Sig.	0.073	0.067	0.069	0.083	0.072	0.077	0.081	0.070	0.076	0.079	0.066	0.075

CO: Cost; LC: Load (carrying) capacity; RE: Repeatability; MMI: Man-Machine Interface; PF: Programming flexibility; MTS: Maximum tip speed; MC: Memory capacity; MR: Manipulator reach; VR: Velocity ratio; DF: Degree of freedom; VSC: Vendor's service contract; PA: Positioning accuracy.

In the criteria assessment questionnaire, the experts used a 5-point Likert scale to determine the significance of each criterion. On a 5-point scale, 3 would be the midpoint and, thus, 3 were tested as the mean. The null hypothesis in a one-sample t-test indicates the insignificance of the proposed criterion. If this hypothesis is rejected, it can be claimed that the criterion is significant when it comes to robot evaluation, and decision-makers should pay sufficient attention to it. Table 4 details the results of the test with Sig. = 5%.

**Table 4:** Hypothesis test for the effectiveness of criteria.

Criterion	One-sample t-test					
	T	Df	Sig.	MD	95% confidence	
					L	U
CO	2.783	47	.000	4.351	4.06	4.65
LC	3.533	47	.000	4.649	4.40	4.90
RE	4.659	47	.000	4.649	4.44	4.86
MMI	3.607	47	.000	4.216	3.95	4.48
PF	6.320	47	.000	4.703	4.50	4.91
MTS	1.733	47	.060	3.703	-1.55	1.86
MC	1.656	47	.075	3.432	-2.22	1.65
MR	1.501	47	.068	3.378	-1.14	2.62
VR	9.259	47	.000	3.892	3.62	4.16
DF	1.127	47	.070	3.514	-1.33	2.70
VSC	1.815	47	.065	3.459	-0.26	3.66
PA	1.656	47	.078	3.459	-1.27	2.65

As seen in Table 4, of the 12 initial criteria, 6 were eliminated due to their t statistic being lower than 1.96 and their significance level higher than 0.05. Therefore, the null hypothesis of their ineffectiveness cannot be rejected. Accordingly, 6 criteria were deemed important by the field experts when it comes to selecting industrial robots.

### 3.2 FUZZY best-worst method (FBWM)

In the third stage, the importance of the identified criteria will be determined. These criteria and their weights can be used to rank the potential industrial robots. According to BWM – introduced by Rezaei (2015)– the best and the worst criteria are identified first by the decision-maker, followed by pairwise comparisons conducted between each of these two criteria (best and worst) and the other criteria (Ramezani et al., 2019). “A MaxiMin problem is then formulated and solved to determine the weights of different criteria. The salient features of the proposed method, compared to the existing multi-criteria decision making (MCDM) methods, are: (1) it requires fewer comparison data; (2) it leads to more consistent comparisons, which means that it produces more reliable results (Rezaei, 2015).” The FBWM is executed in 5 steps (Guo and Zhao, 2017‏):

Step 1 Build the decision criteria system. In this step, the criteria {C1, C2, …, Cn} are considered that should be used to arrive at a decision.

Step 2 Determining the best (e.g., most desirable, most important) and the worst (e.g., least desirable, least important) criteria. In this step, the decision-maker identifies the best and the worst criterion in general and no comparison is made at this stage.

Step 3 Execute the fuzzy reference comparisons for the best criterion. The resulting fuzzy Best-to-Others vector would be: ${\tilde{A}}_{B}=\left({\tilde{a}}_{B1},{\tilde{a}}_{B2},\ldots ,{\tilde{a}}_{Bn}\right)$, where ${\tilde{a}}_{Bj}$ indicates the fuzzy preference of the best criterion over criterion j, and it is clear that ${\tilde{a}}_{BB}=\left(1,1,1\right)$.

Step 4 Execute the fuzzy reference comparisons for the worst criterion. The resulting fuzzy Others-to-Worst vector would be ${\tilde{A}}_{W}=\left({\tilde{a}}_{1W},{\tilde{a}}_{2W},\ldots ,{\tilde{a}}_{nW}\right)$, where ${\tilde{a}}_{iW}$ indicates the preference of the criterion j over the worst criterion and it is clear that ${\tilde{a}}_{WW}=\left(1,1,1\right)$.

Step 5 Finding the optimal fuzzy weights (${\tilde{W}}_{1}^{*},{\tilde{W}}_{2}^{*},\ldots ,{\tilde{W}}_{n}^{*}$). The optimal fuzzy weight for the criteria is the one where, for each pair of $\frac{{\tilde{W}}_{B}}{{\tilde{W}}_{j}}$, and $\frac{{\tilde{W}}_{j}}{{\tilde{W}}_{w}}$, we have $\frac{{\tilde{W}}_{B}}{{\tilde{W}}_{j}}={\tilde{a}}_{Bj}$, and $\frac{{\tilde{W}}_{j}}{{\tilde{W}}_{w}}={\tilde{a}}_{jw}$. To satisfy these conditions for all j, a solution should be found where the maximum absolute differences $\left|\frac{W_{B}}{W_{j}}-a_{Bj}\right|$, and $\left|\frac{W_{j}}{W_{w}}-a_{jw}\right|$ for all j is minimized. The optimization problem to determine the optimal weight of the criteria ($W_{1}^{*},W_{2}^{*},\ldots ,W_{n}^{*}$) is presented as the model (1):

(1) $$\begin{array}{l} Min\;\max_{j}\left\{\left|\frac{W_{B}}{W_{j}}-a_{Bj}\right|,\;\left|\frac{W_{j}}{W_{w}}-a_{jw}\right|\right\}\;\\ s.t:\\ \sum_{j=1}^{n}W_{j}=1,\;\\ W_{j}\geq 0\;,\;\;for\;all\;j \end{array}$$

Then, model (1) turns into the following optimization problem with nonlinear constraints.

(2) $$\begin{array}{l} \text{Min }\tilde{\text{ξ}}\;\\ s.t:\\ \left|\frac{{\tilde{\text{W}}}_{\text{B}}}{{\tilde{\text{W}}}_{\text{j}}}-{\tilde{\text{a}}}_{\text{Bj}}\right|\;\leq \;\tilde{\text{ξ}}\;,\;for\;all\;j\\ \left|\frac{{\tilde{\text{W}}}_{\text{j}}}{{\tilde{\text{W}}}_{\text{w}}}-{\tilde{\text{a}}}_{\text{jw}}\right|\leq \;\tilde{\text{ξ}}\;,\;for\;all\;j\\ \sum_{\text{j}=1}^{\text{n}}\text{R}\left({\tilde{\text{W}}}_{\text{j}}\right)=1\;\\ l_{j}^{w}\leq m_{j}^{w}\leq u_{j}^{w}\\ l_{j}^{w}\geq 0\\ \text{j}=1,2,\ldots ,\text{n} \end{array}$$

Where $\tilde{\text{ξ}}=\left(l_{j}^{w},m_{j}^{w},u_{j}^{w}\right)$. Considering $l_{j}^{w}\leq m_{j}^{w}\leq u_{j}^{w}$, we suppose ${\tilde{\text{ξ}}}^{\text{∗}}=\left(k^{*},k^{*},k^{*}\right),\;\;k^{*}\leq l^{\text{ξ}}$, then nonlinear model (2) can turn into the model (3):

(3) $$\begin{array}{l} Min\;{\tilde{\xi }}^{*}\;\\ s.t:\\ \left|\frac{\left(l_{B}^{w},l_{B}^{w},l_{B}^{w}\right)}{\left(l_{j}^{w},l_{j}^{w},l_{j}^{w}\right)}-\left(l_{Bj},m_{Bj},u_{Bj}\right)\right|\;\leq \;\left(k^{*},k^{*},k^{*}\right)\;,\;for\;all\;j\\ \left|\frac{\left(l_{j}^{w},l_{j}^{w},l_{j}^{w})\right)}{\left(l_{W}^{w},l_{W}^{w},l_{W}^{w}\right)}-(l_{jW},m_{jW},u_{jW})\right|\leq \;\left(k^{*},k^{*},k^{*}\right)\;,\;for\;all\;j\\ \sum_{\text{j}=1}^{\text{n}}\text{R}\left({\tilde{\text{W}}}_{\text{j}}\right)=1\;\\ l_{j}^{w}\leq m_{j}^{w}\leq u_{j}^{w}\\ l_{j}^{w}\geq 0\\ \text{j}=1,2,\ldots ,\text{n} \end{array}$$

By solving model (3), the optimal weights ($W_{1}^{*},W_{2}^{*},\ldots ,W_{n}^{*}$) are obtained.

To determine the weight of the criteria using the FBWM, first, a customized questionnaire was devised, and distributed among 35 experts. Next, based on the opinions of the respondent experts, the most and the least important criteria were established. In the next step, the Best-to-Others preference vector was determined. To do this, all 35 experts were asked to specify their most preferred criterion compared with the other criteria. Afterward, the Others-to-Worst preference vector was also determined. The process of determining the latter was the same as that of the Best-to-Others vector. In the end, the optimization problem was expanded based on Model (3) of the FBWM. After solving the model above using the computer software MATLAB, the final weights of the criteria were organized in Table 5 as follows:

**Table 5:** Criteria weights for industrial robot selection.

Criterion	CO	LC	RE	MMI	PF	VR
W	0.1686	0.2128	0.2002	0.1217	0.1462	0.1524

CO: Cost; LC: Load (carrying) capacity; RE: Repeatability; MMI: Man-Machine Interface; PF: Programming flexibility; VR: Velocity ratio.

### 3.3 Preference Ranking Organization Method for Enrichment Evaluations (PROMETHEE)

Assuming that there are some collections of options among which we intend to choose; if there are k criteria which are effective in decision-making, for every possibility of a∈A, *f j* (*a*) indicates the j^th^ index's value in option *a* (Brans et al., 1986). The process of ranking is accomplished in 3 steps:

First step: $P_{j}$ preference function is allocated to every j criteria. $P_{j}\left(a,b\right)$ is calculated for every couple of options. This amount is changing between 0 and 1. If the relationship of $f_{j}\left(a\right)=f_{j}\left(b\right)$ can be made, the amount of $P_{j}\left(a,b\right)$ becomes zero and through the rising of $f_{j}\left(a\right)-f_{j}\left(b\right)$, this amount will be also increased. And when the difference becomes much enough, the amount of $P_{j}\left(a,b\right)$ will be 1 (Brans, J.P., et al., 1986). Various diagrams can be assumed for $P_{j}$ function, which is dependent upon modeling state of the j^th^ criterion. PROMETHEE method suggests six types of preference functions to the decision-maker. It should be noticed that a $w_{j}$ variable of scale is considered for every $f_{j}$ criterion.

Second step: the general priority of π(a,b) for every alternative a over alternative b is calculated. The more π(a,b) is, the more alternative a has preference. π(a,b) is calculated as follows (Brans and De Smet, 2016):

(4) $$\pi \left(a,b\right)=\sum_{j=1}^{k}W_{j}P_{j}\left(a,b\right),\;\;\left(\sum_{j=1}^{k}W_{j}=1\right)$$

Third step: π(a,b) is the indication of the degree of priority of alternative a over alternative *b*. To evaluate the general preference capability of alternative a over other options, the positive ranking flow is calculated (Chou et al., 2004):

(5) $$\varphi^{+}\left(a\right)=\frac{1}{n-1}\sum_{x\varepsilon A}\pi \left(a,x\right)$$

It indicates the capability of the alternative a. The largest $\varphi^{+}\left(a\right)$ is the best alternative. Preference amount of other alternatives over alternative *a*, which is called the negative ranking flow is calculated as follows:

(6) $$\varphi^{-}\left(a\right)=\frac{1}{n-1}\sum_{x\varepsilon A}\pi \left(x,a\right)$$

This flow makes it clear how much other alternatives take priority over alternative a. The smallest $\varphi^{-}\left(a\right)$ is the best alternative. Therefore, through a calculation of positive and negative flowage, a partial ranking can be achieved (ranking in PROMETHEE I). To have a complete ranking of alternatives, pure flowage of ranking should be defined for every alternative (ranking in PROMETHEE II):

(7) $$\varphi \;\left(a\right)=\varphi^{+}\left(a\right)-\varphi^{-}\left(a\right)$$

This flow is the result of the balance between positive and negative ranking flows. The larger net flow is the superior alternative (De Leeneer and Pastijn, 2002).

The problem of selecting the most suitable industrial robot for the given pick-n-place operation is solved using proposed framework. This framework could be used for every industrial robot for material handling, packing, transportation, polishing, welding, and grading.

There are two approaches to demonstrate and validate the proposed procedure. The first approach uses real data for evaluation. In many of the papers that have applied this approach, the process of index selection has not been carried out and several specific robots with the same data have been evaluated in all articles (Bhangale et al., 2004; Chatterjee et al., 2010; Rao et al., 2011; Athawale and Chakraborty, 2011; Chakraborty, 2011; etc.). In the second approach, a numerical example can be used. Since the proposed model is not limited to a particular type of robot, the second approach is used in this study.

The first step of ranking allocates a preference function to every criterion. Among six functions that exist, one function has been determined for every criterion based on the kind of data and decision-makers’ judgment. The threshold of indifference and threshold of strict preference should be appointed for some functions. We use the example mentioned in (Lui et al., 2014) to characterize a manufacturing company that requires a robot to perform a material handling task and that the prospective robot buyer, at most, can afford to spend $ 75,000. After a task analysis, it has been identified that the desired load capacity should be at least 30 lb. and the repeatability should be within 0.6 mm. Moreover, man-machine interface, programming flexibility, and velocity ratio are also considered as important evaluation criteria. After the initial selection, four robots (A1, A2, A3, and A4) that satisfy the requirements of the particular problem are chosen for further evaluation. In order to select the most suitable robot, the decision-maker needs to collect the desired information. This information has been presented in Table 6. Through determining the mentioned issues, the preferred amount of the alternative concerning each other can be calculated based on paired comparisons.

**Table 6:** Decision matrix.

Criterion	CO	LC	RE	MMI	PF	VR
Criterion type	C	B	B	B	B	B
Weight	0.1686	0.2128	0.2002	0.1217	0.1462	0.1524
preference function	V-Shape	V-Shape	V-Shape	V-Shape	V-Shape	V-Shape
q	-	-	-	-	-	-
p	10,000	20	0.25	0.20	0.200	0.100
A1	73,000	68	0.40	0.52	0.656	0.125
A2	70,000	60	0.40	0.59	0.754	0.75
A3	68,000	50	0.13	0.41	0.761	0.100
A4	64,000	30	0.60	0.48	0.732	0.55

CO: Cost; LC: Load (carrying) capacity; RE: Repeatability; MMI: Man-Machine Interface; PF: Programming flexibility; VR: Velocity ratio.

Table 7 show the final ranking of industrial robots.

**Table 7:** Ranking robots.

Rank	Alternative	Ranking Flows		
		φ+	φ-	φ
2	A1	0.3333	0.2307	0.1027
1	A2	0.3018	0.1662	0.1356
4	A3	0.1798	0.4096	-0.2271
3	A4	0.3124	0.3236	-0.0112

## 4 Conclusion

The final goal of industrial robots evaluation and selection problem is to select a proper robot that is magnificently adaptable to the company's requirements. There are many robots with distinct specifications, and choosing the best alternative concerning various conflicting criteria can be complicated. There is a need for a simple and logical scientific method or mathematical tool to guide user organizations in taking a proper decision. In this paper, a proper procedure applied to determine the relative importance of decision making criteria and ranking of alternative robots. This work developed an integrated MCDM approach combing the FBWM and PROMETHEE method for the selection of the optimal industrial robots. The methodology developed in this paper helps decision-makers in selecting a suitable robot by considering both conflicting quantitative and qualitative selection criteria in real-life applications. Moreover, a numerical example has demonstrated analytically the computational process of the proposed method. The above findings confirm the effectiveness of the model, that even though it uses a relatively simple mathematical formulation and straightforward operation, it is capable of solving complex multi-attribute decision problems, incorporating both quantitative and qualitative factors. The decision model exhibited here for choosing robots is a general method. It can be employed for making the best decision in other fields of engineering and management problems.

## Declarations

### Author contribution statement

Mahdi Nasrollahi, Javaneh Ramezani & Mahmoud Sadraei: Conceived and designed the analysis; Analyzed and interpreted the data; Contributed analysis tools or data; Wrote the paper.

### Funding statement

The authors received no specific funding for this work.

### Competing interest statement

The authors declare no conflict of interest.

### Additional information

No additional information is available for this paper.
